# Disruption of purine *de novo* synthesis pathway impairs membrane homeostasis, intracellular survival, and virulence of *Brucella melitensis*

**DOI:** 10.3389/fmicb.2025.1721961

**Published:** 2025-12-16

**Authors:** Guangyu Yang, Mengsi Li, Yang Li, Simin Chen, Jing Qu, Jihong Li, Shaohui Wang, Yanqing Bao, Jingjing Qi, Yafeng Dou, Mingxing Tian

**Affiliations:** 1 Shanghai Veterinary Research Institute, Chinese Academy of Agricultural Sciences, Shanghai, China; 2 Hubei Key Laboratory of Tumor Microenvironment and Immunotherapy, China Three Gorges University, Yichang, China; 3 Department of Pathology, The Second People's Hospital of China Three Gorges University, Yichang, China

**Keywords:** *Brucella*, *de novo* purine synthesis, membrane homeostasis, intracellular survival, virulence

## Abstract

**Introduction:**

Brucellosis is a significant zoonotic infectious disease caused by *Brucella*. As a facultative intracellular pathogen, the ability of *Brucella* to acquire essential nutrients within host cells is critical for its intracellular survival and pathogenicity. Previous studies have indicated that disruption of the *de novo* purine biosynthesis pathway significantly attenuates the virulence of *Brucella*, although the underlying mechanisms remain incompletely understood.

**Methods:**

Using *Brucella melitensis* M5 as the parental strain, we constructed a deletion mutant and a complemented strain of the key purine biosynthesis gene *purD* (encoding phosphoribosylamine–glycine ligase) via homologous recombination. Bacterial growth and stress sensitivity were assessed. Membrane permeability was evaluated using propidium iodide (PI) and 1-N-phenylnaphthylamine (NPN), while lipid biosynthesis was examined via Nile red staining. Additionally, intracellular survival was tested in cell infection models, and overall virulence was evaluated in a mouse model.

**Results:**

Deletion of *purD* disrupted *de novo* purine synthesis and impaired bacterial growth. The mutant exhibited increased sensitivity to the anionic detergent sodium dodecyl sulfate, reduced membrane permeability, and altered lipid biosynthesis. Furthermore, the *purD* mutant showed significantly reduced ability to invade and survive within host cells, primarily due to insufficient purine acquisition. In mice, the mutant displayed reduced spleen and liver colonization, with diminished splenomegaly and granuloma formation.

**Discussion:**

This study demonstrates that *de novo* purine biosynthesis is critical for *Brucella* membrane integrity, intracellular survival, and full virulence. These findings highlight this pathway as a promising target for developing attenuated vaccines and novel antimicrobials against brucellosis.

## 1 Introduction

Brucellosis is one of the most common zoonotic diseases with a worldwide distribution, primarily infecting cattle, sheep, pigs, dogs, and humans. Infection in animals can lead to various clinical manifestations such as arthritis, abortion, and infertility, while human patients often present with undulant fever, night sweat, and joint pain. The widespread prevalence of brucellosis poses a major challenge to the development of global livestock industries and public health security, particularly in developing countries (Boschiroli et al., 2001). The disease's insidious nature and multi-host characteristics make it difficult to achieve comprehensive surveillance and effective control (Qureshi et al., 2023). It has been recognized by the World Health Organization (WHO) as a neglected disease of major zoonotic importance (Franc et al., 2018).

Brucellosis is caused by infection with *Brucella*, a Gram-negative, facultative intracellular pathogen. Unlike many other pathogenic bacteria, *Brucella* lacks typical virulence factors such as exotoxins, capsules, and secreted proteases. Its pathogenicity primarily stems from its ability to survive and proliferate within host cells (Seleem et al., 2008). After entering host cells, *Brucella* resides within a membrane-bound vacuole, called *Brucella*-containing vacuole (BCV). This vacuole fuses with endosomes to form an endosomal BCV (eBCV), which subsequently establishes limited contact with lysosomes before fusing with endoplasmic reticulum exit sites to become a replicative BCV (rBCV). Following intracellular replication, the bacterium partially interacts with autophagosomes to form an autophagic BCV (aBCV), ultimately leading to fusion with the plasma membrane and bacterial egress (Celli, 2019). The intracellular survival of *Brucella* relies on a range of sophisticated regulatory mechanisms, including the inhibition of apoptosis, interference with antigen presentation, suppression of inflammatory pathway activation, and reprogramming of macrophage function (Martirosyan et al., 2011; Xavier et al., 2013). Among these, nutrient acquisition plays a critical role in supporting the intracellular replication of *Brucella*. For example, glucose uptake was crucial for increased replication of *B. abortus* in alternatively activated macrophages and for chronic infection (Xavier et al., 2013); Erythritol and erythronate utilization promote *Brucella* proliferation in placenta of pregnant animals, inducing abortion (Letesson et al., 2017; Yin et al., 2023). Nucleotides, which are critical for bacterial functions such as DNA replication and energy storage, also play a vital role in bacterial virulence.

Nucleotides are not only essential building blocks for DNA and RNA synthesis, but their derivatives also play critical roles in numerous cellular functions, such as energy metabolism, signal transduction, and protein synthesis (Goncheva et al., 2022). Purine biosynthesis occurs via two pathways: *de novo* synthesis and salvage synthesis. Among these, the *de novo* purine synthesis pathway, as one of the fundamental metabolic processes, plays a critical role in the regulation of bacterial virulence (Goncheva et al., 2022). Generally, wild-type bacteria utilize precursor molecules (such as phosphoribosyl pyrophosphate, glutamine, glycine, etc.) through the *de novo* pathway to produce purines and purine nucleotides to support their growth requirements (Kilstrup et al., 2005). Disruption of *de novo* purine synthesis affects multiple virulence-associated processes, including the synthesis and secretion of virulence factors, iron acquisition, and signal transduction, thereby impairing bacterial pathogenicity (Song et al., 2009; Jenkins et al., 2011). Deletion of key genes in the purine synthesis pathway leads to purine nucleotide deficiency, which attenuates bacterial virulence. Mutations in purine biosynthetic pathways have been extensively studied in many pathogens and are often implicated in the regulation of bacterial virulence, as demonstrated in *Francisella tularensis* (Quarry et al., 2007), *Bacillus anthracis* (Jenkins et al., 2011), and *Staphylococcus aureus* (Connolly et al., 2017).

In *Brucella*, to our knowledge, Drazek et al. first demonstrated that the deletion of N5-carboxyaminoimidazole ribonucleotide mutase (PurE) in *B. melitensis* 16M significantly attenuated intracellular growth within macrophages (Drazek et al., 1995). Subsequently, Crawford and Cheville independently reported that deletion of the *purE* gene markedly reduced the virulence of *B. melitensis* 16M in mice and goats (Cheville et al., 1996; Crawford et al., 1996). Later, multiple studies utilizing random transposon mutagenesis screens identified several mutants of purine synthesis genes in *Brucella* that exhibited impaired replication within host cells. For example, *B. suis* mutants with disruptions in *purD* and *purF* showed reduced proliferation in THP-1 cells (Kohler et al., 2002), while Mariner transposon insertions inactivating *purH, purK, purS*, and *purQ* in *B. melitensis* impaired its survival in J774.A1 cells (Wu et al., 2006). Additionally, transposon sequencing revealed that *purA, purE*, and *purS* are essential for the intracellular replication of *B. abortus* in RAW264.7 cells (Sternon et al., 2018). Besides, Alcantara et al. reported that an intact purine biosynthesis pathway is essential for the virulence of *B. abortus* 2308 in mice (Alcantara et al., 2004). Furthermore, Truong et al. showed that deletion of *purD* and *purF* further attenuated the residual virulence of the attenuated vaccine strain *B. abortus* RB51 (Truong et al., 2015). Collectively, these studies indicate that the *de novo* purine synthesis pathway plays a crucial role in *Brucella* virulence. However, the specific biological mechanisms and phenotypic alterations through which purine synthesis defects lead to virulence attenuation in *Brucella* remain poorly understood.

In this study, using *B. melitensis* M5 as the parental strain, we successfully constructed a *purD* gene deletion mutant and its complemented strain via bacterial homologous recombination. *In vitro* growth curve assays confirmed that the deletion of *purD* completely disrupts the *de novo* purine synthesis pathway in *B*. *melitensis*. Through stress tolerance assays, fluorescent dye uptake experiments, and cell infection models, we demonstrated that impairment of the *de novo* purine synthesis pathway compromises membrane homeostasis, reduces the ability to invade host cells, and diminishes intracellular survival. Mouse infection experiments further verified that the purine synthesis deficiency attenuates the capacity of *Brucella* to cause lethal infection, colonize the spleen, induce splenomegaly, and form hepatic granulomas. Therefore, our findings indicate that the *de novo* purine synthesis pathway is essential for *Brucella* to maintain membrane homeostasis and exert full virulence. This study provides new insights for the development of live attenuated vaccines based on purine synthesis defects and potential therapeutic targets against brucellosis.

## 2 Results

### 2.1 The *purD* deletion disrupted the purine *de novo* synthesis pathway in *B. melitensis*

The *purD* gene in *Brucella* spans 1284 bp and encodes a 428-amino acid phos-phoribosylamine–glycine ligase, which catalyzes the conversion of 5-phosphoribosylamine (PRA) to glycinamide ribonucleotide (GAG)—a critical step in the *de novo* purine biosynthesis pathway. Using *B. melitensis* strain M5 as the parental strain, we constructed a *purD* deletion mutant (Δ*purD*) and a complemented strain (C*purD*). In the Δ*purD* mutant, a 1165-bp fragment representing 90.7% of the open reading frame was deleted (Supplementary Figure S1A). For the C*purD* strain, the full-length *purD* gene, along with its putative promoter and terminator regions, was integrated as a single copy into the intergenic region between *glmS* and *recG* genes on chromosome II using the mini-Tn7 transposon system, enabling stable genetic complementation (Supplementary Figure S1B).

**Figure 1:**
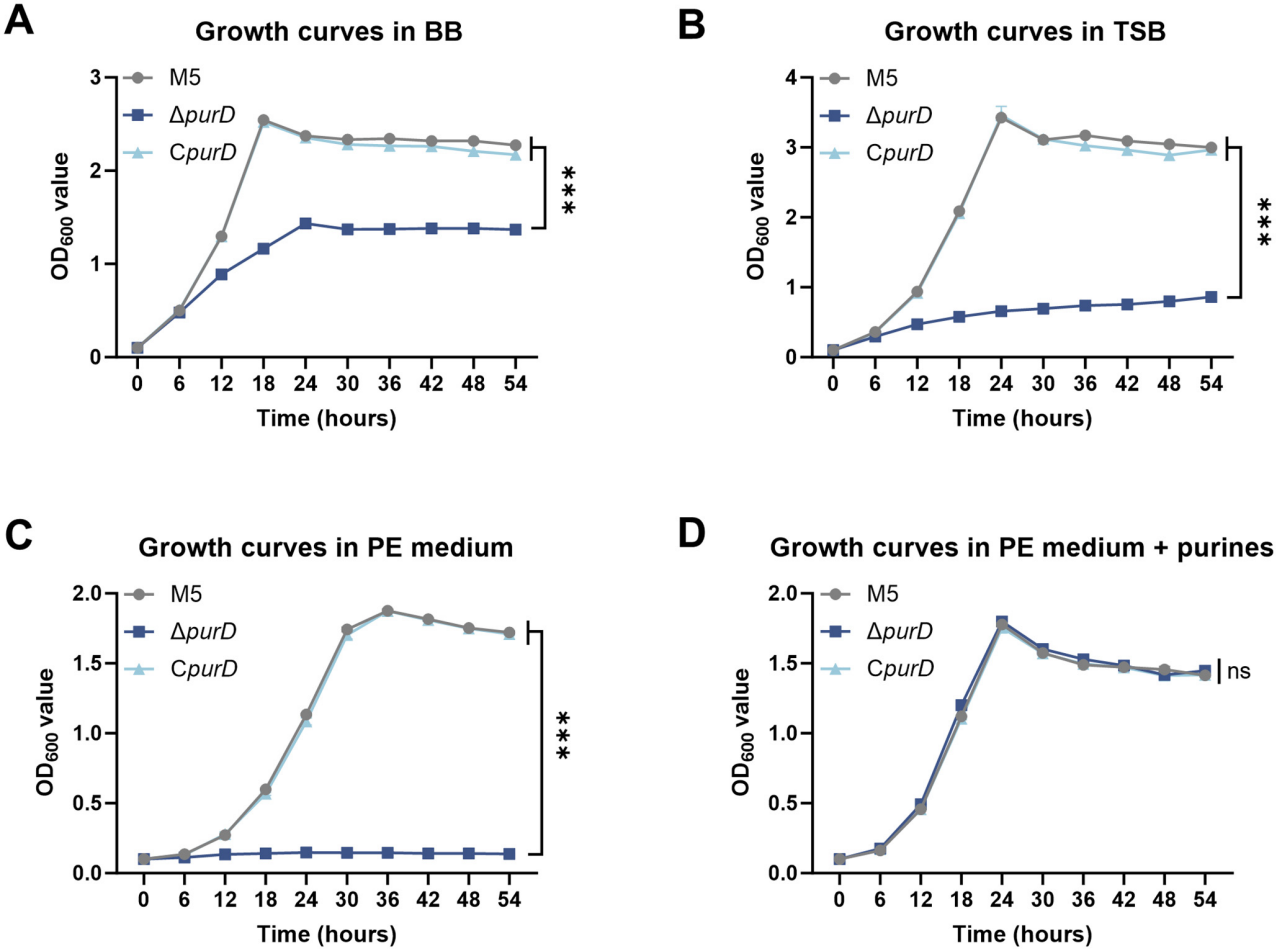
Determination of growth curves of *Brucella* and its derivative strains. **(A)** Growth curves measured in *Brucella* Broth (BB); **(B)** Growth curves measured in Tryptic Soy Broth (TSB); **(C)** Growth curves measured in the chemically defined medium PE (Plommet's medium with 2 g/L erythritol); **(D)** Growth curves measured in PE supplemented with 1 mM guanine, 1 mM adenine, and 1 mM hypoxanthine. Statistical significance was determined using two-way ANOVA (***p < 0.001; ns, not significant).

To assess whether *purD* deletion affects the *in vitro* growth capacity of *Brucella* M5, we compared the growth of the parental M5, Δ*purD* mutant, and C*purD* complemented strains in nutrient-rich *Brucella* Broth (BB) and Tryptic Soy Broth (TSB). As shown in Figures 1A, B, the Δ*purD* mutant exhibited significantly impaired growth in both media compared to the parental strain, whereas the C*purD* strain restored growth to a level comparable to M5, indicating that *purD* is critical for normal *in vitro* growth.

To determine whether this growth impairment resulted from defective purine acquisition, we evaluated bacterial growth in a chemically defined Plommet's medium supplemented with erythritol (PE) (Machelart et al., 2020), which lacks exogenous purines. The Δ*purD* mutant failed to grow in PE medium, while the C*purD* strain recovered growth to the level of M5 (Figure 1C). Supplementation of PE medium with 1 mM guanine, adenine, and hypoxanthine fully restored the growth of the Δ*purD* mutant to levels comparable to both the parental and complemented strains (Figure 1D).

Together, these findings demonstrate that the growth defect of the Δ*purD* mutant is due to a disruption in *de novo* purine synthesis, confirming that PurD is essential for purine biosynthesis in *B. melitensis*.

### 2.2 Impaired purine acquisition alters membrane homeostasis and affects lipid synthesis in *Brucella*

**Figure 2:**
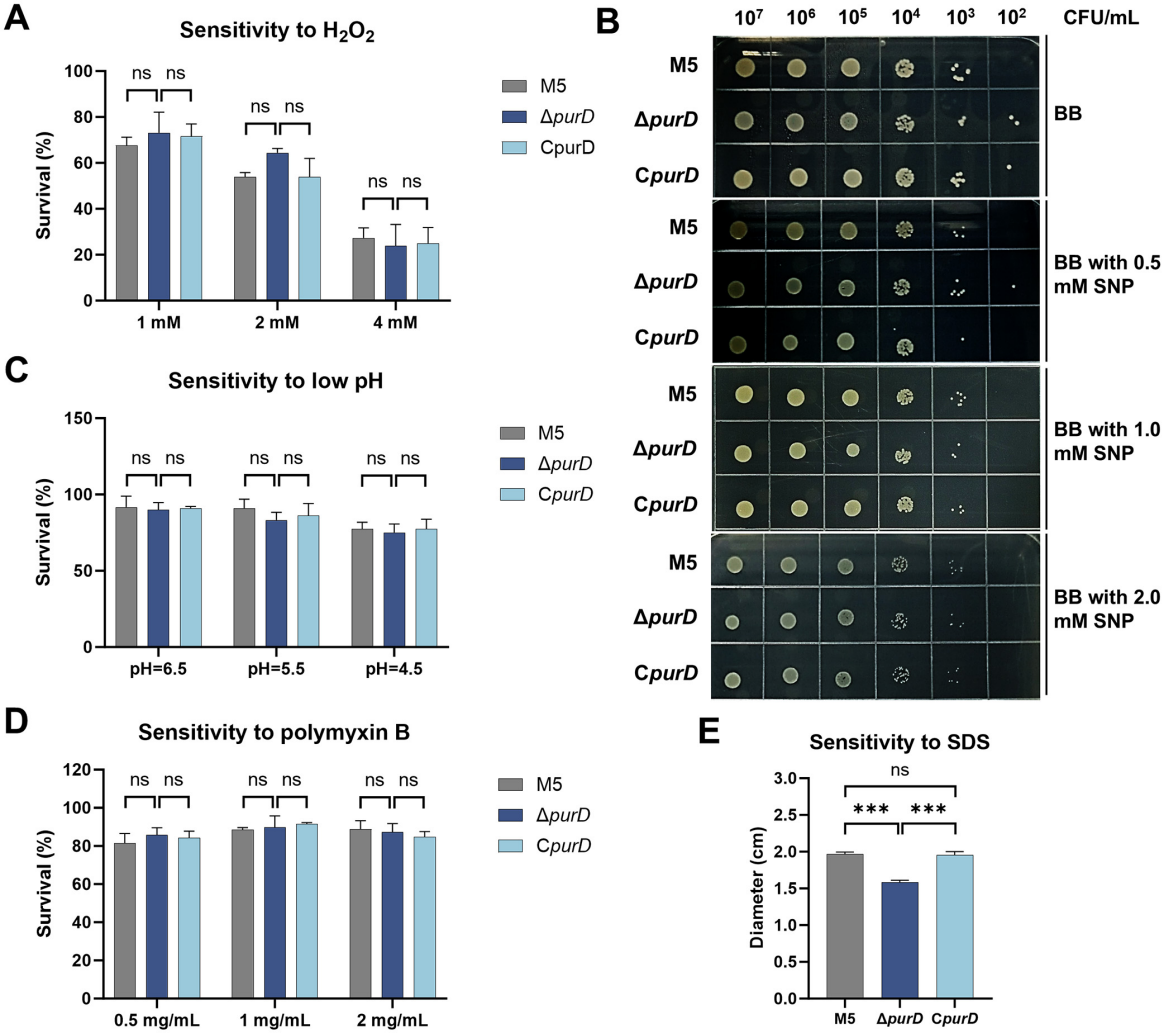
Sensitivity of *Brucella* strains to stress factors. **(A)** Hydrogen peroxide; **(B)** Sodium nitroprusside (SNP); **(C)** Acidic *Brucella* Broth (BB) medium; **(D)** Polymyxin B; **(E)** Sodium dodecyl sulfonate (SDS). Statistical significance was determined using one-way or two-way ANOVA (***p < 0.001; ns, not significant).

To determine whether *purD* deletion affects the stress resistance of *Brucella*, we subjected the parental M5, Δ*purD* mutant, and complemented C*purD* strains to a series of stress susceptibility assays. Tolerance to oxidative stress was evaluated using hydrogen peroxide; nitrosative stress was assessed with sodium nitroprusside (SNP, a nitric oxide donor); acid tolerance was tested under acidic conditions; and susceptibility to cationic antimicrobial peptides and membrane integrity were evaluated using polymyxin B and sodium dodecyl sulfate (SDS), respectively. As shown in Figure 2, no significant differences were observed among the strains in response to hydrogen peroxide (Figure 2A), SNP (Figure 2B), acidic stress (Figure 2C), or polymyxin B (Figure 2D), indicating that *purD* deletion does not affect resistance to these stressors. Notably, the Δ*purD* mutant exhibited a significantly smaller inhibition zone in the SDS disk diffusion assay compared to the parental and complemented strains (Figure 2E). Since SDS disrupts bacterial membranes, this result suggests altered membrane structure in the mutant. However, Western blot analyses of both biotin-labeled *Brucella* membrane proteins and lipopolysaccharide (LPS) using monoclonal antibodies demonstrated that the *purD* deletion did not alter the expression profiles of membrane proteins or LPS (Supplementary Figures S2, S3).

**Figure 3:**
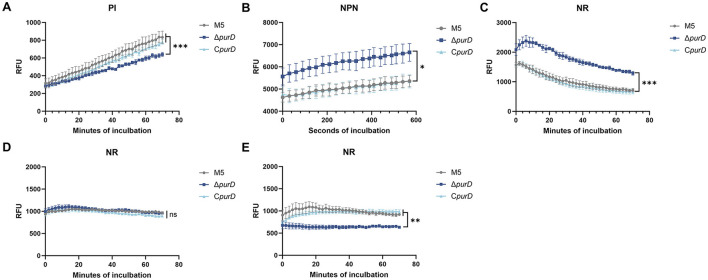
Fluorescent dye uptake of *Brucella* and its derivative strains. **(A)** The uptake of propidium iodide (PI) in bacteria cultivated in BB medium. **(B)** The uptake of 1-N-phenylnaphthylamine (NPN) in bacteria cultivated in BB medium. **(C)** The uptake of Nile Red (NR) in bacteria cultivated in BB medium. **(D)** The uptake of NR in bacteria cultivated in PE medium. **(E)** The uptake of NR in bacteria cultivated in PE medium supplemented with 1 mM guanine, 1 mM adenine, and 1 mM hypoxanthine. Statistical significance was determined using two-way ANOVA (*p < 0.05; **p < 0.05; ***p < 0.001; ns, not significant).

To further investigate membrane alterations, we assessed membrane integrity using propidium iodide (PI), a DNA-intercalating fluorescent dye that only penetrates cells with compromised membranes. Dynamic fluorescence measurements revealed significantly lower PI fluorescence in the Δ*purD* mutant than in the M5 and C*purD* strains (Figure 3A), indicating reduced membrane permeability. We next employed the hydrophobic probe N-phenyl-1-naphthylamine (NPN), which exhibits enhanced fluorescence upon integration into phospholipid membranes. The Δ*purD* mutant showed markedly higher NPN fluorescence compared to the parental and complemented strains (Figure 3B), suggesting increased membrane hydrophobicity or altered composition. Based on these observations, we hypothesized that lipid synthesis might be upregulated in the mutant. To test this, we used Nile red (NR), a dye whose fluorescence intensifies in hydrophobic lipid-rich environments. NR staining revealed significantly higher fluorescence in the Δ*purD* mutant than in both the parental and complemented strains (Figure 3C), supporting enhanced lipid accumulation.

To further investigate the impact of purine metabolism defects on lipid synthesis in *Brucella*, we performed NR staining on *Brucella* cultured in both PE medium and PE medium supplemented with purines. The results showed that under PE medium conditions, there was no significant difference in fluorescence intensity between the Δ*purD* mutant and the parental or complemented strains (Figure 3D). However, under purine-supplemented PE medium conditions, the fluorescence intensity of the Δ*purD* mutant was significantly lower than that of the parental and complemented strains (Figure 3E).

These experimental findings indicate that *purD* deletion affects membrane homeostasis in *Brucella*. Under nutrient-rich conditions, *purD* deletion enhances lipid synthesis in *Brucella*, whereas under nutrient-limited conditions, it attenuates lipid synthesis.

### 2.3 Impairment of *de novo* purine synthesis attenuates the ability of *Brucella* to invade host cells and survive intracellularly

**Figure 4:**
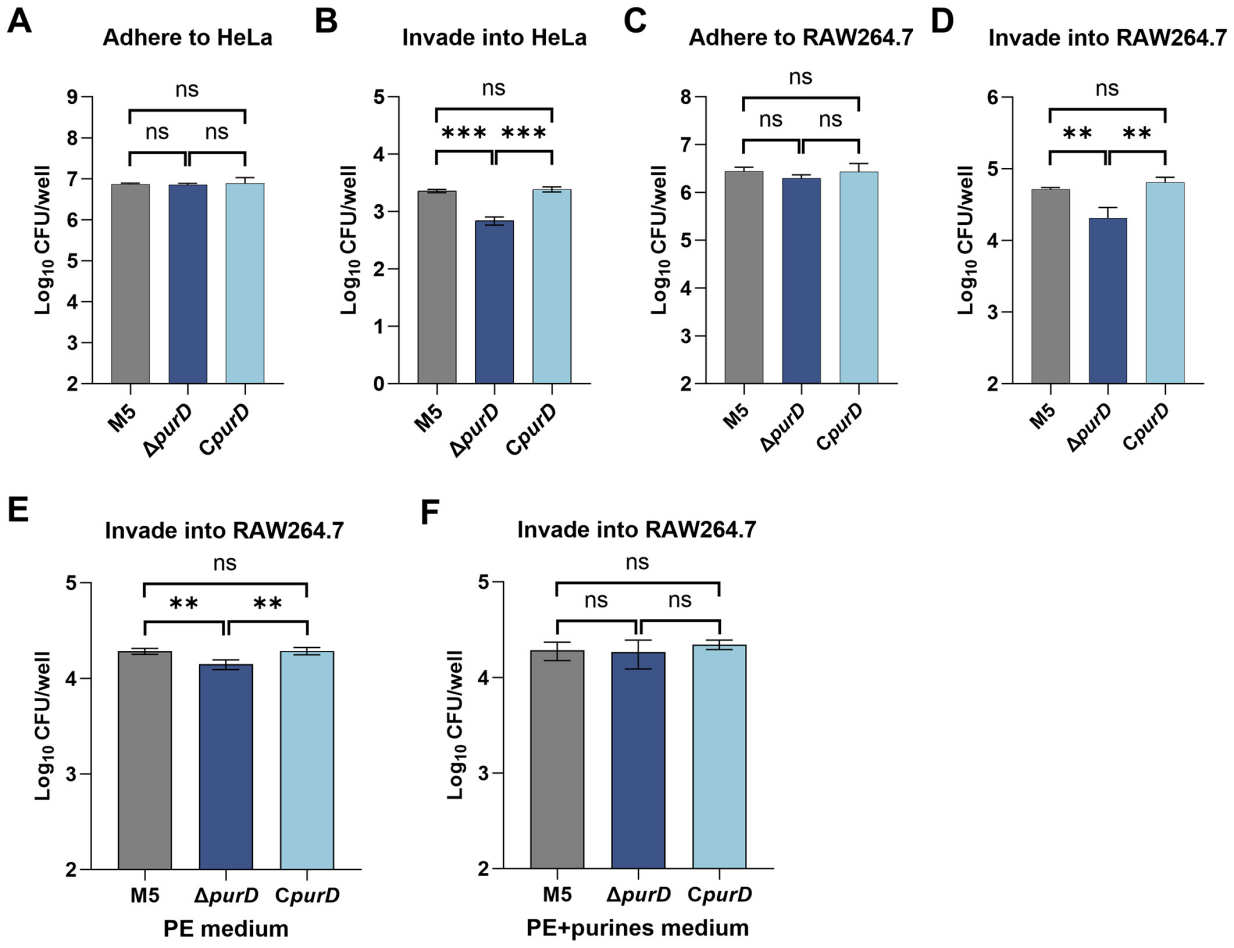
Adhesion and invasion of host cells by *Brucella* and its derivative strains. Bacteria were cultured in BB, PE, or purine-supplemented PE medium prior to infection. **(A)** Adhesion and **(B)** invasion of HeLa cells by bacteria grown in BB. **(C)** Adhesion and **(D)** invasion of RAW264.7 cells by bacteria grown in BB. **(E)** Invasion of RAW264.7 cells by bacteria grown in PE. **(F)** Invasion of RAW264.7 cells by bacteria grown in PE supplemented with 1 mM guanine, 1 mM adenine, and 1 mM hypoxanthine. Statistical significance was determined using one-way ANOVA (**p < 0.01; ***p < 0.001; ns, not significant).

Adhesion to and invasion of host cells, as well as intracellular survival, are crucial for the pathogenicity of *Brucella* (von Bargen et al., 2012). To evaluate whether purine synthesis deficiency affects the infectivity of *Brucella*, we conducted a detailed comparison of the abilities of the parental strain M5, the Δ*purD* mutant, and the complemented strain C*purD* to adhere to and invade human epithelial-like HeLa cells and murine monocytic macrophage RAW264.7 cells. Infection assays revealed that, in both HeLa and RAW264.7 cells, the Δ*purD* mutant showed no difference in adhesion capability compared to the parental and complemented strains (Figures 4A, C), but exhibited a significantly reduced ability to invade host cells (Figures 4B, D). To investigate whether this invasion defect was attributable to purine deficiency, we evaluated the cell invasion capacity of bacteria cultured in either PE medium or PE medium supplemented with purines. The results showed that under PE medium conditions, the Δ*purD* mutant exhibited a significantly reduced ability to invade RAW264.7 cells compared to the parental and complemented strains (Figure 4E). In contrast, when cultured in purine-supplemented PE medium, the invasion capacity of the Δ*purD* mutant showed no significant difference from that of the parental and complemented strains (Figure 4F). This result indicates that purine synthesis deficiency impairs the ability of *Brucella* to invade host cells.

**Figure 5:**
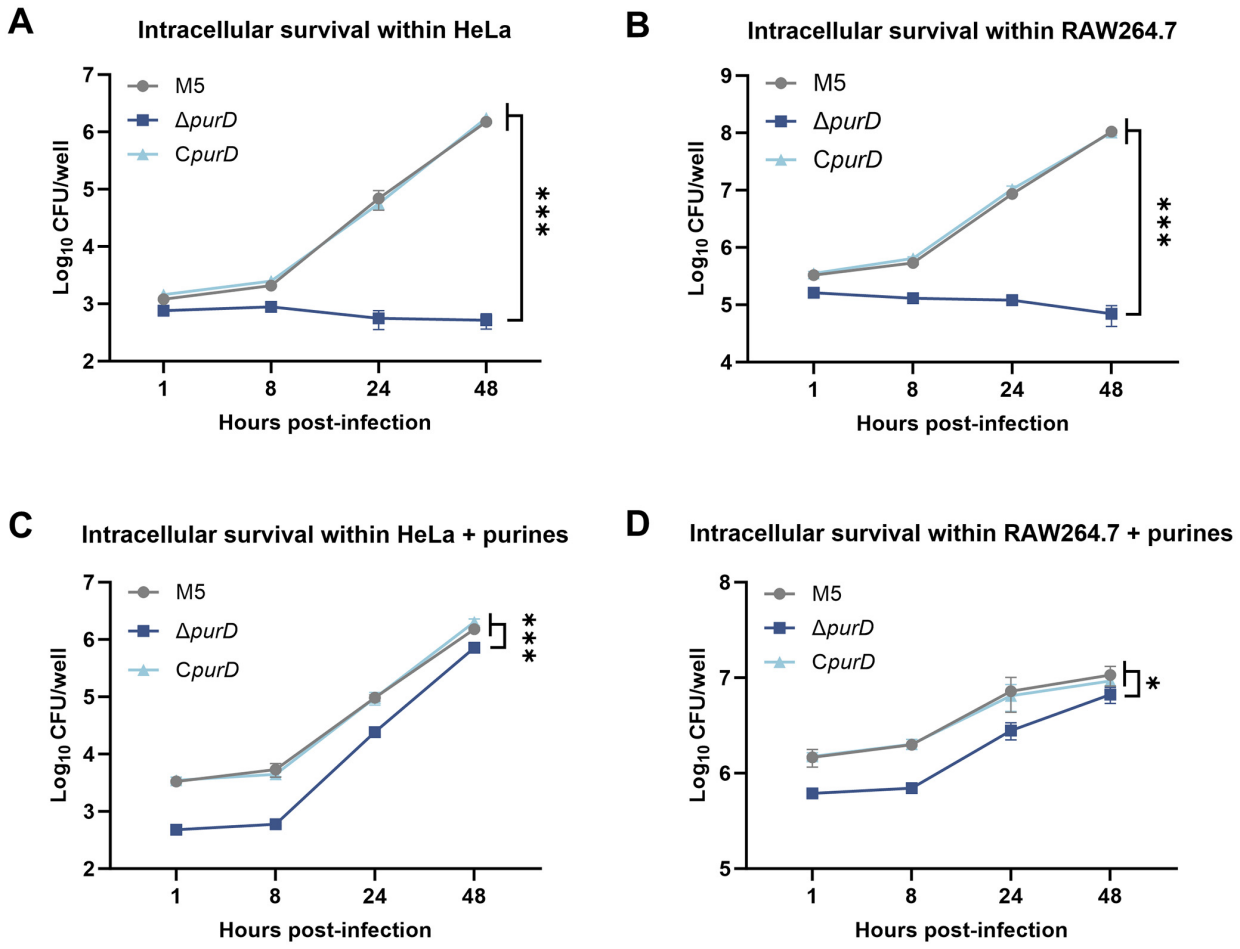
Intracellular survival of *Brucella* and its derivative strains. **(A)** Intracellular survival in HeLa cells; **(B)** Intracellular survival in RAW264.7 cells; **(C)** Intracellular survival in HeLa cells with exogenous supplementation of 1 mM guanine, 1 mM adenine, and 1 mM hypoxanthine; **(D)** Intracellular survival in RAW264.7 cells with exogenous supplementation of 1 mM guanine, 1 mM adenine, and 1 mM hypoxanthine. Statistical significance was determined using two-way ANOVA (*p < 0.05; ***p < 0.001).

Intracellular survival assays demonstrated that, compared to the parental strain, the Δ*purD* mutant failed to proliferate within both HeLa and RAW264.7 cells, indicating a significantly attenuated intracellular survival capacity. Complementation with the *purD* gene fully restored the intracellular proliferation ability of the mutant, making it comparable to that of the parental strain (Figures 5A, B). To determine whether the intracellular proliferation defect of the Δ*purD* mutant was due to insufficient purine acquisition, we supplemented the cell culture medium with 1 mM guanine, 1 mM adenine, and 1 mM hypoxanthine during the intracellular survival assay. The results showed that the Δ*purD* mutant regained the ability to proliferate intracellularly, although its survival level remained significantly lower than that of the parental and complemented strains (Figures 5C, D). This difference was primarily attributed to the lower initial bacterial load of the Δ*purD* mutant invading the cells at 1 h post-infection (h.p.i.) compared to the parental and complemented strains.

**Figure 6:**
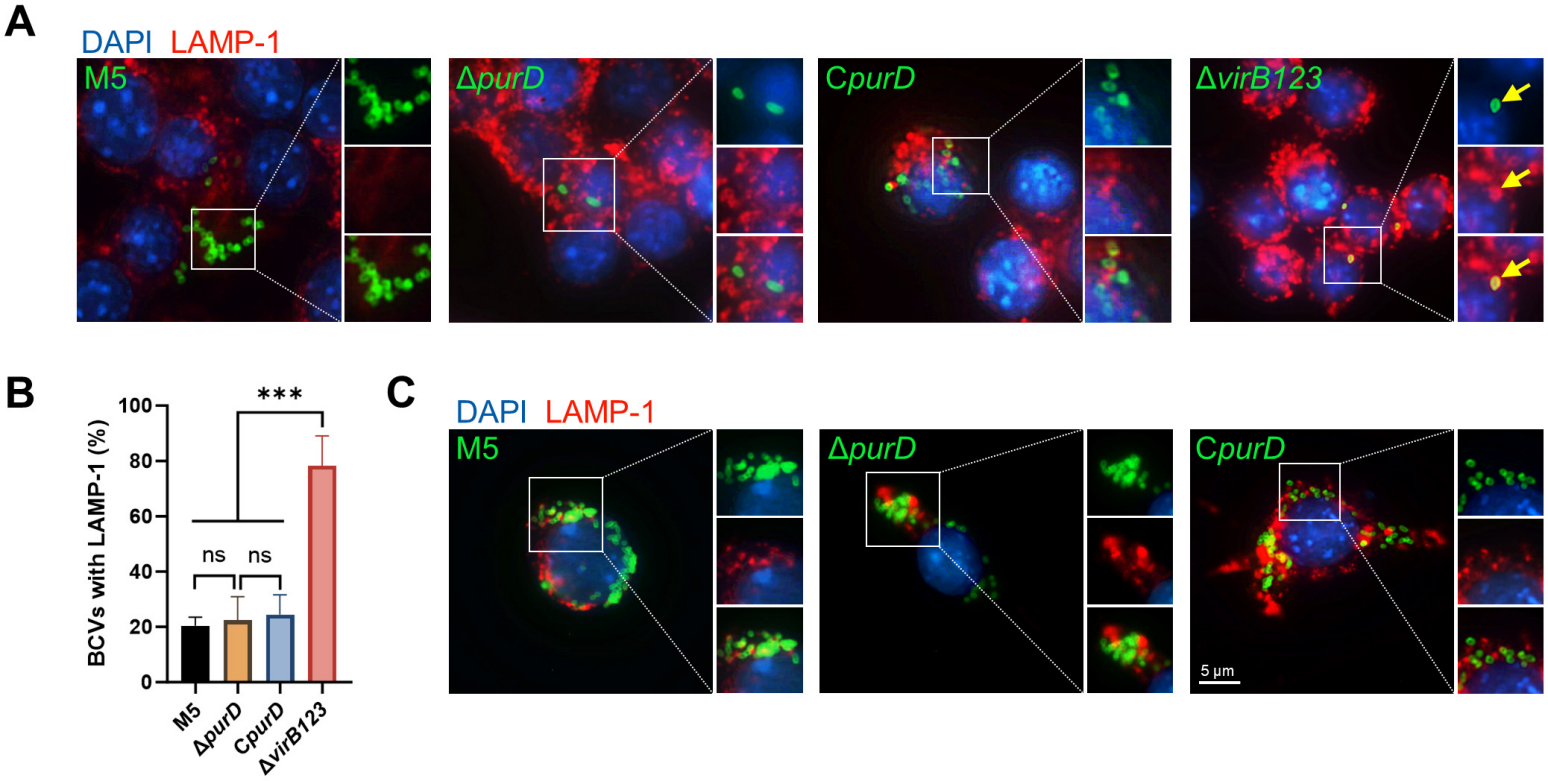
Colocalization analysis of *Brucella* and its derivative strains with the lysosomal marker LAMP-1. **(A)** Representative images of colocalization between *Brucella* and LAMP-1 by indirect immunofluorescence staining at 24 h post-infection (h.p.i) in RAW264.7 cells; **(B)** Statistical analysis of the percentage of BCVs (*Brucella*-containing vacuoles) colocalized with LAMP-1; **(C)** Representative images of colocalization between *Brucella* and LAMP-1 by indirect immunofluorescence staining at 24 h.p.i. in RAW264.7 cells exogenously supplemented with 1 mM guanine, 1 mM adenine, and 1 mM hypoxanthine. Statistical significance was determined using one-way ANOVA (***p < 0.001; ns, not significant).

For *Brucella* to achieve intracellular replication, it must establish a replicative niche and form mature rBCVs (Celli, 2019). A hallmark of mature rBCVs is their ability to evade fusion with lysosomes (Celli et al., 2003). In this study, we investigated whether the inability of the Δ*purD* mutant to proliferate intracellularly was due to a failure in forming mature rBCVs. To test this hypothesis, we examined the colocalization of BCVs with the lysosomal marker LAMP-1 in RAW264.7 cells at 24 h.p.i. in the absence of exogenous purine supplementation. As shown in Figure 6A, although the Δ*purD* mutant was unable to replicate efficiently intracellularly, the BCVs it formed showed no significant colocalization with LAMP-1, a characteristic shared with the replicative parental strain M5 and the complemented strain C*purD*. In contrast, the positive control strain Δ*virB123* (lacking a functional type IV secretion system) exhibited pronounced colocalization with LAMP-1.

We further quantified the percentage of BCVs colocalizing with LAMP-1. As shown in Figure 6B, the colocalization percentages for the parental strain M5, the Δ*purD* mutant, and the complemented strain were approximately 20%, all significantly lower than that of the positive control strain Δ*virB123* (approximately 80%), but with no significant differences among the three. Additionally, we performed indirect immunofluorescence assays to observe the intracellular replication of the Δ*purD* mutant in the presence of exogenous purines. As shown in Figure 6C, exogenous purine supplementation resulted in obvious proliferation of the Δ*purD* mutant, consistent with the intracellular survival results mentioned above. Moreover, the BCVs formed by the intracellular Δ*purD* mutant displayed a similar pattern to those of the M5 and C*purD* strains, showing no significant colocalization with LAMP-1.

All these experimental data demonstrate that purine synthesis deficiency impairs the ability of *Brucella* to invade host cells. Once inside the host cell, *Brucella* with impaired purine synthesis cannot acquire sufficient exogenous purines from the host to support its intracellular survival. Thus, *de novo* purine synthesis is essential for the intracellular survival of *Brucella*.

### 2.4 Impairment of the *de novo* purine synthesis pathway significantly attenuates the virulence of *Brucella*

**Figure 7:**
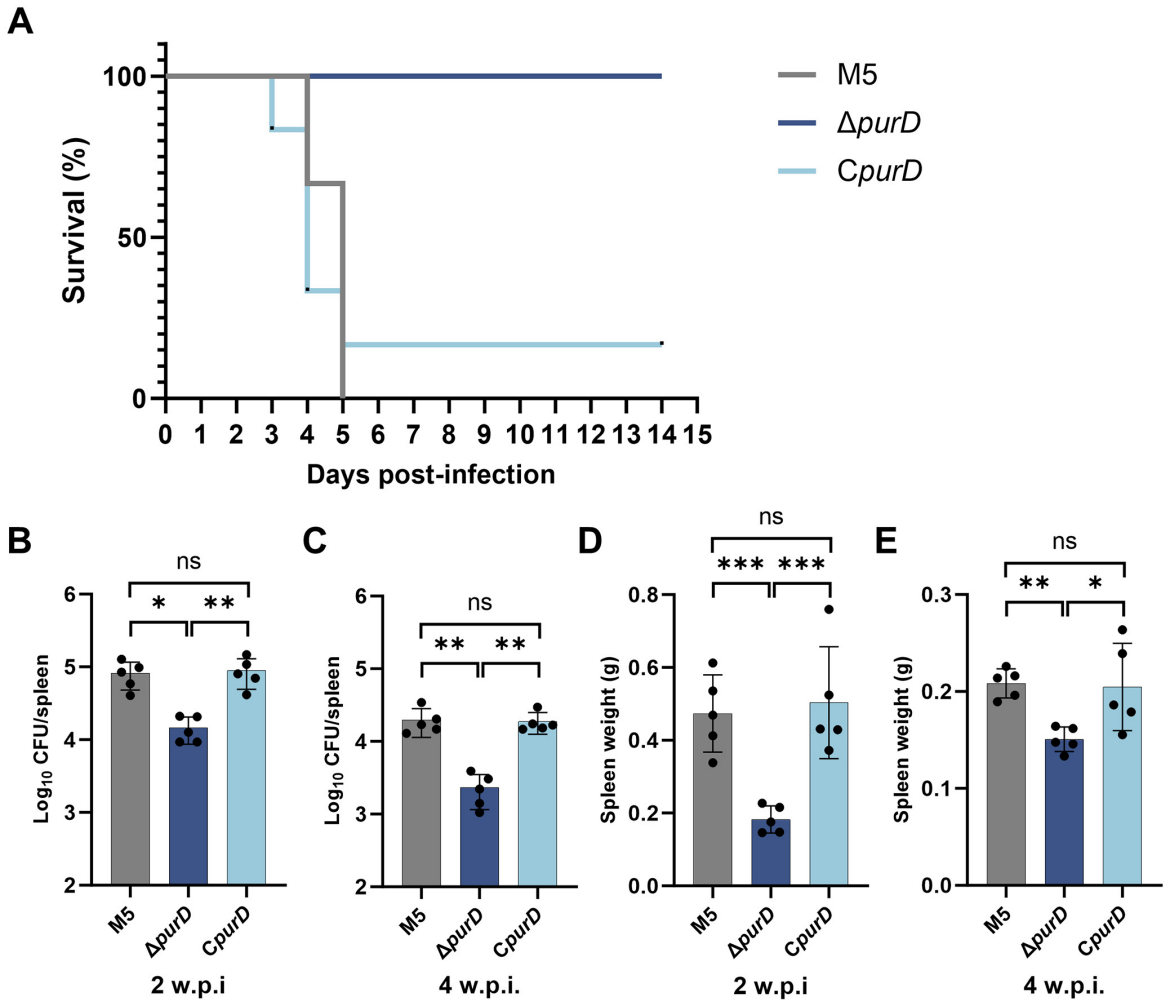
Pathogenicity analysis of *Brucella* infection in mice. **(A)** Survival curves of mice within 14 days after intraperitoneal injection with 2.5 × 10^8^ CFU of *Brucella*; **(B, C)** Bacterial loads in the spleen at 2 and 4 weeks post-infection (w.p.i.) with 1 × 10^6^ CFU of *Brucella*; **(D, E)** Spleen weight at 2 and 4 w.p.i. Statistical significance was determined using one-way ANOVA (*p < 0.05; **p < 0.01; ***p < 0.001; ns, not significant).

To evaluate the impact of purine synthesis on *Brucella* virulence, we assessed the survival rates and splenic bacterial loads in mice following intraperitoneal infection with high and low inoculum doses of the parental strain M5, the Δ*purD* mutant, and the complemented strain C*purD*. When mice were challenged with a high dose (2.5 × 10^8^ CFU), all six mice infected with the Δ*purD* mutant survived the 14-day observation period. In contrast, all six mice infected with the parental strain M5 succumbed to infection within 5 days (Figure 7A). Only one of six mice infected with the complemented strain C*purD* survived until day 14 (Figure 7A). These results indicate that the deletion of *purD* abolishes the lethal capacity of *Brucella* in mice.

Following infection with a low dose (1 × 10^6^ CFU), the splenic bacterial loads were quantified at 2 and 4 weeks post-infection (w.p.i.). The bacterial burden in the spleens of mice infected with the Δ*purD* mutant was significantly lower than that in mice infected with the parental strain M5. The complemented strain C*purD* restored the bacterial load to a level comparable to the parental strain (Figures 7B, C). Spleen weight, an indicator of splenomegaly induced by *Brucella* infection, was also significantly reduced in mice infected with the Δ*purD* mutant compared to those infected with the parental or complemented strains (Figures 7D, E). This demonstrates that *purD* deletion severely impairs the ability of *Brucella* to colonize the spleen and cause splenomegaly.

**Figure 8:**
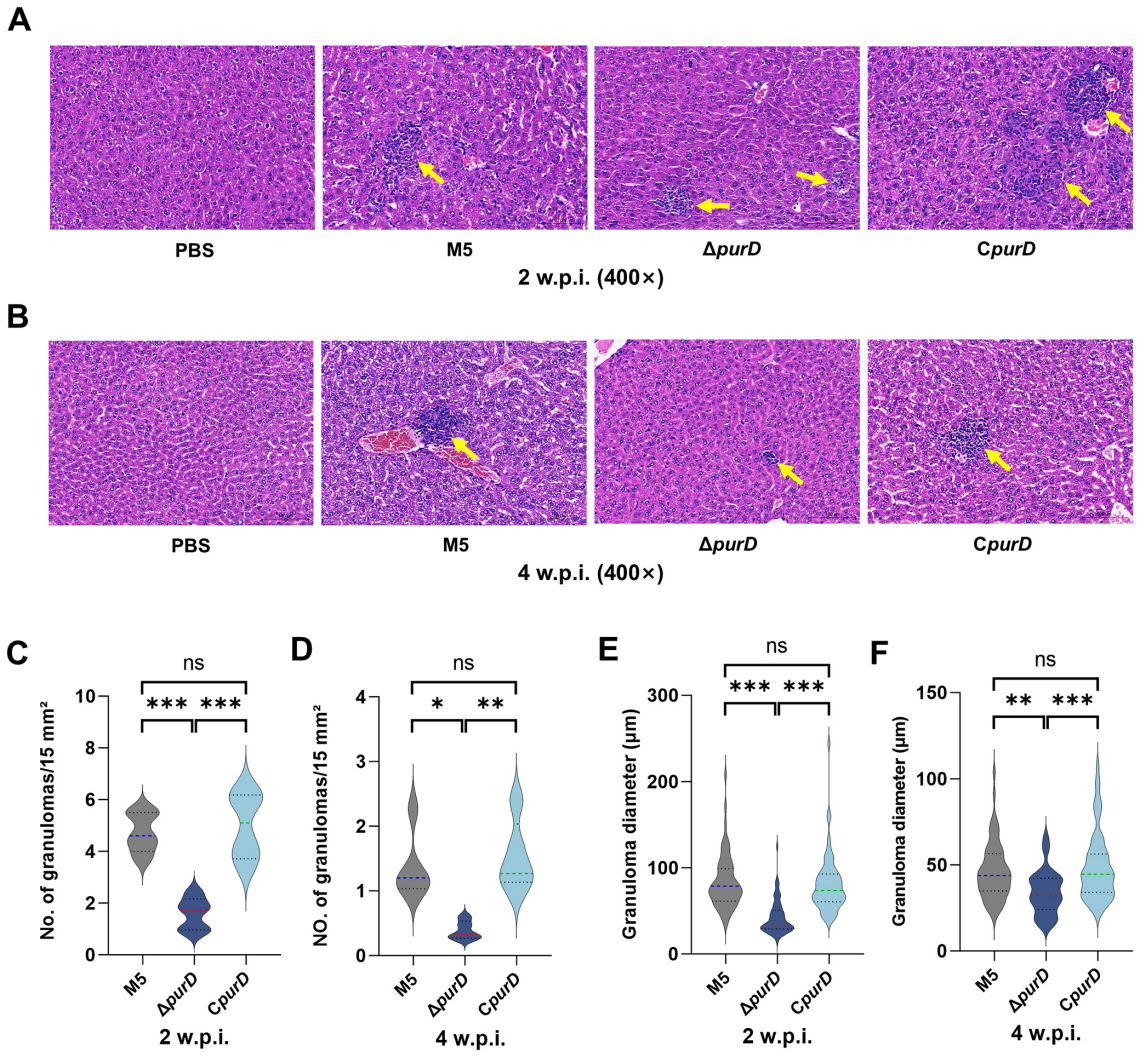
Histopathological analysis of liver sections from mice infected with *Brucella* and its derivative strains. **(A, B)** H&E staining of liver tissues at 2 and 4 weeks postinfection (400 × magnification); **(C, D)** Number of granulomas per 15 mm^2^ area; **(E, F)** Diameter of granulomas. Statistical significance was determined using one-way ANOVA (*p < 0.05; **p < 0.01; ***p < 0.001; ns, not significant).

To further assess the pathogenicity of the Δ*purD* mutant, histopathological changes in the livers of low-dose infected mice were examined. As shown in Figure 8A, at 2 w.p.i., the parental strain M5 and the complemented strain C*purD* induced the formation of large, distinct granulomas in the liver. In contrast, the granulomas formed in response to the Δ*purD* mutant were notably smaller. At 4 w.p.i., prominent granulomas were still evident in the livers of mice infected with the parental and complemented strains, while only small granulomas were observed in the Δ*purD* group (Figure 8B). No significant pathological changes were detected in the livers of PBS-inoculated control mice.

For quantitative analysis of hepatic histopathology, the number of granulomas per 15 mm^2^ of liver tissue was randomly counted at 2 and 4 w.p.i. (Figures 8C, D). The number of granulomas in mice infected with the Δ*purD* mutant was significantly lower than in those infected with the parental or complemented strains. This difference was particularly pronounced at 4 weeks, where the average number of granulomas per 15 mm^2^ was 0.39 in the Δ*purD* group, compared to 1.36 in the parental group and 1.52 in the complemented group. Furthermore, the diameters of the granulomas were measured (Figures 8E, F). At both 2 and 4 w.p.i., the granulomas in the Δ*purD*-infected mice were significantly smaller than those in the parental and complemented groups, indicating that *purD* deletion impairs the ability of *Brucella* to induce granuloma formation in the liver.

In all, these data demonstrate that a defect in *de novo* purine synthesis significantly attenuates the pathogenicity of *Brucella* in mice.

## 3 Discussion

This study aimed to investigate the impact of the *de novo* purine biosynthesis pathway on the virulence of *Brucella*. Our results demonstrate that disruption of this pathway not only compromises bacterial membrane stability and alters lipid synthesis but also significantly impairs the ability of *Brucella* to invade host cells and proliferate intracellularly, ultimately leading to attenuated virulence in mice. This study systematically establishes that an intact *de novo* purine biosynthesis pathway is essential for *B. melitensis* to maintain full virulence.

The *de novo* purine biosynthesis pathway initiates from 5-phosphoribosyl-1-pyrophosphate (PRPP) and proceeds through multiple enzymatic steps to generate inosine monophosphate (IMP), the common precursor of all purine nucleotides such as AMP and GMP (Goncheva et al., 2022). Among these, PurD catalyzes the second step, converting 5-phosphoribosylamine (PRA) to glycinamide ribonucleotide (GAR) (Goncheva et al., 2022). In this study, the Δ*purD* mutant failed to grow in purine-free PE medium, indicating that the deletion of *purD* completely disrupted the *de novo* purine synthesis in *Brucella*. However, when guanine, adenine, and hypoxanthine were supplemented to the PE medium, the growth defect of the Δ*purD* mutant was rescued, demonstrating that *Brucella* can synthesize the required purine nucleotides via the salvage pathway. Furthermore, these findings collectively demonstrate that *Brucella* can obtain purine nucleotides through both *de novo* synthesis and salvage pathways. Notably, even in nutrient-rich media such as TSB or BB, the growth of the Δ*purD* mutant was significantly impaired compared to the parental and complemented strains. This phenotype is consistent with observations in *purD* and *purF* mutants of *B. abortus* RB51 (Truong et al., 2015), suggesting that *Brucella* heavily relies on its endogenous purine synthesis capacity to sustain optimal growth, even in environments with relatively abundant purine sources.

Although purines are essential metabolites for bacterial growth, defects in their synthesis typically attenuate virulence indirectly by inhibiting growth. However, growing evidence suggests that aberrant purine metabolism may also directly affect various virulence-related phenotypes. For instance, purine-auxotrophic *Escherichia coli* and *Salmonella* Typhimurium exhibit reduced intestinal colonization capacity and impaired resistance to oxidative stress in macrophages, respectively (Mantena et al., 2008; Vogel-Scheel et al., 2010). In rough-type *B. abortus* RB51, deletion of *purD* or *purF* also enhances susceptibility to hydrogen peroxide (Truong et al., 2015). However, in the smooth-type *B. melitensis* used in this study, we did not observe a significant change in hydrogen peroxide sensitivity in the Δ*purD* mutant. This discrepancy may be attributed to differences in bacterial surface structures, such as the O-antigen (Martin-Martin et al., 2011). Interestingly, we found for the first time that a defect in purine synthesis markedly reduced bacterial sensitivity to SDS. As an anionic detergent, SDS primarily exerts its bactericidal effect by disrupting membrane structure (Woldringh and van Iterson, 1972); this phenomenon has not been previously reported. Moreover, recent studies suggest a link between purine metabolism and antibiotic susceptibility: point mutations in several purine biosynthesis genes (e.g., *purA, purD*) in *E. coli* enhance sensitivity to carbenicillin but not to gentamicin (Lubrano et al., 2025), further implying that the status of purine metabolism may influence cell membrane/wall integrity.

To assess membrane structural integrity, we subjected membrane proteins to biotin labeling and detected LPS using monoclonal antibodies, followed by Western blot analysis. The results indicated no significant differences in membrane protein or LPS expression in the Δ*purD* mutant (Supplementary Figures S2, S3). However, PI staining indicated increased membrane permeability in the mutant. Concurrently, enhanced fluorescence of the hydrophobic dye NPN and the lipophilic dye NR suggested significantly elevated lipid accumulation. Notably, *Brucella* cultured in the chemically defined medium PE vs. PE supplemented with purines exhibited distinct lipid synthesis patterns. In PE medium, the Δ*purD* mutant showed no significant lipid alterations. However, when grown in purine-supplemented PE medium, the Δ*purD* strain displayed reduced lipid synthesis, indicating that impaired purine acquisition modulates lipid metabolism in the mutant. To our knowledge, no previous studies have reported that purine deficiency enhances lipid synthesis in *Brucella*. Intriguingly, in *Bacillus subtilis*, purine deficiency lowers GTP levels, thereby alleviating CodY-mediated repression of lipid synthesis and promoting fatty acid accumulation (Brinsmade and Sonenshein, 2011). Furthermore, the deletion of *purD* led to impaired growth of *Brucella* (Figure 1). We hypothesize that in nutrient-rich medium, abundant carbon sources may be preferentially channeled into lipid storage, whereas under nutrient-limited conditions, exogenous purines stimulated the growth of the Δ*purD* mutant, thereby directing carbon flux toward growth at the expense of lipid storage. The precise mechanism by which *purD* deletion influences lipid synthesis in *Brucella* warrants further investigation.

The pathogenicity of *Brucella* heavily relies on its ability to invade cells and survive intracellularly. This study found that the Δ*purD* mutant exhibited significantly reduced invasion efficiency in both HeLa and RAW264.7 cells, consistent with trends observed in *purE* deletion mutants of *B. melitensis* 16M and *purD/purF* deletion mutants of *B. abortus* RB51 (Drazek et al., 1995; Truong et al., 2015). Notably, when cultured in nutrient-rich BB medium, the Δ*purD* mutant exhibited enhanced lipid synthesis (Figure 3C) and a markedly reduced ability to invade host cells (Figures 4B, D). In contrast, under nutrient-limited PE medium conditions, lipid synthesis levels in the mutant were comparable to those of the parental strain (Figure 3D). Although the invasion capacity remained impaired under these conditions (decreased by 0.14 log_10_; Figure 4E), the reduction was substantially less severe than that observed in nutrient-rich medium (0.4 log_10_ decrease; Figure 4D). Moreover, supplementation of PE medium with purines led to a significant decrease in lipid synthesis (Figure 3E) and restored the invasion ability of the Δ*purD* mutant to a level similar to that of the parental strain (Figure 4F). Collectively, these results imply that elevated lipid synthesis may critically compromise the host cell invasion capacity of the Δ*purD* mutant, although the precise mechanistic basis remains to be elucidated.

After invading host cells, *Brucella* depends on its Type IV Secretion System (T4SS) to evade lysosomal degradation and to interact with the endoplasmic reticulum-Golgi compartments to establish a replicative niche (von Bargen et al., 2012; Celli, 2019). In this study, although the Δ*purD* mutant completely lost its ability to replicate intracellularly, it still effectively escaped lysosomal degradation. Furthermore, exogenous purine supplementation fully restored its replication defect, indicating that this phenotype primarily results from purine starvation rather than impaired intracellular trafficking or niche establishment. According to Deghelt et al., *Brucella* enters a growth-arrested G1 phase within 4–6 h.p.i., with replication typically initiating after 8 h.p.i. (Deghelt et al., 2014). We hypothesize that purine demand is low during the G1 phase, but upon entering the replication phase, the Δ*purD* mutant fails to replicate due to its inability to meet purine requirements through *de novo* synthesis. This result also indirectly suggests that purines within the host cell may not be readily accessible to *Brucella*, further underscoring the critical role of the *de novo* synthesis pathway in intracellular survival.

*In vivo* experiments demonstrated that the Δ*purD* mutant had a significantly reduced colonization capacity in mouse spleens, consistent with phenotypes observed in purine-auxotrophic mutants of strains 16M, 2308, and RB51 (Crawford et al., 1996; Alcantara et al., 2004; Truong et al., 2015). This study further revealed that infection with the mutant resulted in a significant reduction in both the number and size of granulomas in the liver. In high-dose infection experiments, the lethality of the Δ*purD* mutant was also markedly diminished. These results collectively indicate that host target organs cannot provide sufficient exogenous purines during infection, necessitating *Brucella* to rely on its own synthesis pathway to support proliferation.

Although this study confirms the necessity of the *de novo* purine biosynthesis pathway for the virulence of *B. melitensis* through *purD* deletion, several important questions remain for future investigation: How does purine deficiency specifically affect lipid metabolism? Do deletions of other genes in this pathway cause similar phenotypes? What are the underlying molecular mechanisms? Is this phenomenon conserved across other species within the *Brucella* genus? Future research should focus on the following aspects: systematically constructing deletion mutants of other key genes in the purine pathway to comprehensively assess their impact on lipid metabolism; integrating multi-omics analyses (transcriptomics, metabolomics, proteomics) to elucidate the regulatory network linking purine and lipid metabolism; and conducting comparative validation in other species such as *B*. *suis* to determine the conservation of this mechanism within the genus.

This study not only establishes the central role of *de novo* purine synthesis in *Brucella* pathogenesis—where its disruption leads to severe impairment of intracellular replication and *in vivo* colonization—but also offers new perspectives for developing live attenuated vaccines. Previous studies have shown that attenuated *B. abortus* RB51 Δ*purD*/Δ*purF* and *B. melitensis* M5 Δ*purE*/Δ*purK* mutants still elicit effective immune protection (Truong et al., 2015; Wang et al., 2025). Given that commonly used vaccine strains (e.g., Rev.1, S19) still exhibit residual virulence (Bosseray and Plommet, 1990), constructing attenuated strains based on purine auxotrophy may provide a promising strategy for developing safer and more effective *Brucella* vaccines.

## 4 Materials and methods

### 4.1 Bacterial strains and culture conditions

The attenuated *B. melitensis* vaccine strain M5 (CVCC, Beijing, China), which was derived from the wild-type strain M28, and its derived strains were cultured in BB (HuanKai Microbial, Guangzhou, China) or TSB (Difco, Franklin Lakes, NJ, USA). Solid media were prepared by adding 1.5% agar (Sangon Biotech, Shanghai, China) to the broth to obtain *Brucella* Agar (BA). All *Brucella* strains were incubated at 37 °C under 5% CO_2_. All experiments involving live low virulence *B. melitensis* strains were conducted in a Biosafety Level 2 (BSL-2) facility at the Shanghai Veterinary Research Institute. *E. coli* DH5α (TIANGEN Biotech, Beijing, China) was grown in Luria–Bertani (LB) medium supplemented with 50 μg/mL kanamycin (Sangon Biotech) when necessary. All strains and plasmids used in this study are listed in Table 1.

**Table 1:** Bacterial strains and plasmids used in this study.

**Strains and plasmids**	**Description**	**Sources**
**Strains**		
*B*. *melitensis* M5	Attenuated vaccine strain; smooth phenotype.	CVCC
Δ*purD*	The *purD* deletion mutant deprived from M5.	This study
C*purD*	The *purD* complemented strain.	This study
Δ*virB123*	The *virB1, virB2* and *virB3* deletion mutant	Yin et al., 2024
*E. coli* DH5α	F–, ϕ80d*lacZ* ΔM15, Δ*(lacZYA-argF)*U169, *recA1, endA1, hsdR17(rk–, mk+), phoA, supE44, thi-1, gyrA96, relA1*, λ-	TIANGEN
**Plasmids**		
pKB	Kan^R^; pUC19 derived plasmid containing *sacB* gene.	Liu et al., 2025
pMiniTn7TK	Amp^R^, Kan^R^; mini-Tn7 vector synthesized based on the pUC8T-mini-Tn7T plasmid.	Choi et al., 2005
pHelp1	Amp^R^; expression of Tn7 transposase TnsABCD constructed based on the pTNS2 plasmid.	Choi et al., 2005
pKB-Δ*purD*	pKB plasmid carrying the upstream and downstream homologous arm fragments of the *purD* gene.	This study
pMiniTn7TK-C*purD*	pMiniTn7TK carrying the *purD* gene with its putative promoter and terminator regions	This study

### 4.2 Construction of plasmids and recombinant strains

The suicide plasmid was constructed following a previously described method (Liu et al., 2025). Briefly, genomic DNA of *B. melitensis* strain M5 was used as the template to amplify the upstream (1048 bp) and downstream (1039 bp) fragments of the *purD* gene by PCR with primer pairs purD-UF/UR and purD-DF/DR, respectively. These fragments were then fused using overlap PCR with primers purD-UF and purD-DR, and the resulting product was purified by gel extraction. The purified fusion fragment was ligated into the linearized pKB plasmid using the EasyGeno Assembly Cloning Kit (TIANGEN) and transformed into *E. coli* DH5α competent cells, yielding the plasmid pKB-Δ*purD*.

For the complementation plasmid, the *purD* gene and its predicted promoter region were amplified by PCR with primers CpurD-F and CpurD-R. The gel-purified amplicon was cloned into the *Bam*H I- and *Kp*n I-digested pMiniTn7TK vector using the same cloning kit mentioned above, resulting in the complementation plasmid pMiniTn7TK-C*purD* (Choi et al., 2005). All primers used for PCR in this study are listed in Table 2.

**Table 2:** Primers used in this study.

**Primers**	**Sequence (5^′^-3^′^)**
purD-UF	GGTACCCGGGGATCCTATCGAGCACGAGTATCTGG
purD-UR	CCTCATAGGCACGCGGATCAACAGAACTTTCATGC
purD-DF	AAAGTTCTGTTGATCCGCGTGCCTATGAGGCGCTG
purD-DR	TGCCTGCAGGTCGACCTGACCCTTCTGGTGATCGG
CpurD-F	ATGAGCTCACTAGTGGATCCGATGCCAGAAAGACGGCAGC
CpurD-R	CAAGGCCTTCGCGAGGTACCGCATCTCCCCAAACCGGCAG

The *purD* deletion mutant was constructed using a previously reported *sacB*-based counterselection system (Liu et al., 2025). Briefly, the suicide plasmid pKB-Δ*purD* was electroporated into *Brucella* competent cells that had been washed twice with ice-cold sterile water and resuspended in 10% glycerol. Electroporation was performed at 2.4 kV and 400 Ω. Primary selection was carried out on BA plates containing 50 μg/mL kanamycin to identify integrants. Counterselection was then performed on plates containing 5% sucrose to achieve markerless deletion, resulting in the Δ*purD* mutant strain. To construct the complemented strain, the complementation plasmid pMiniTn7TK-C*purD* was electroporated into the Δ*purD* mutant under the same conditions, with the aid of a helper plasmid pHelp1 (Choi et al., 2005). Transformants were selected on kanamycin-containing BA plates, yielding the complemented strain C*purD*.

### 4.3 Growth curve determination

To determine the effect of *purD* gene deletion on the growth of *Brucella*, this study evaluated the growth of the M5 strain, the Δ*purD* mutant, and the complemented strain C*purD* in nutrient-rich BB and TSB media. The detailed procedure was as follows: M5 and its derivative strains were cultured in BB or TSB to the logarithmic growth phase, and the optical density (OD_600_) of the bacterial suspension was measured and adjusted to 1.0. The bacterial suspension was then inoculated at a 1:10 ratio into fresh BB or TSB medium and incubated at 37°C with shaking at 200 rpm. Every 6 h, the bacterial suspension was collected to measure the OD_600_ value until the bacteria reached the stationary phase. Growth curves were plotted based on the OD_600_ measurements.

To assess the impact of *purD* gene deletion on the *de novo* purine synthesis pathway in *Brucella*, this study also evaluated the growth of the M5 strain, the Δ*purD* mutant, and the complemented strain C*purD* in a modified chemically defined Plommet's medium (PE) (Machelart et al., 2020). The composition of PE medium included 2.3 g/L K_2_HPO4, 3 g/L KH_2_PO4, 0.1 g/L Na_2_S_2_O3, 5 g/L NaCl, 0.2 g/L niacin, 0.2 g/L thiamine, 0.07 g/L pantothenate, 0.5 g/L (NH4)_2_SO4, 0.01 g/L MgSO4, 0.1 mg/L MnSO4, 0.1 mg/L FeSO4, 0.1 mg/L biotin, 1 mM methionine, and 2 g/L meso-erythritol. Since this medium lacks exogenous purines, 1 mM guanine, 1 mM adenine, and 1 mM hypoxanthine was added to the PE medium as required by the experimental design. The bacterial culture conditions and OD_600_ measurements were performed as described above, and growth curves were generated based on the OD_600_ values.

### 4.4 Stress factor susceptibility assay

*Brucella* M5 and its derivative strains were cultured in BB medium to the logarithmic growth phase and adjusted to OD_600_ = 1.0 (approximately 5 × 10^9^ CFU/mL). The bacterial suspension was diluted with PBS to approximately 5 × 10^5^ CFU/mL. Then, 50 μL of the diluted suspension was mixed with either polymyxin B or hydrogen peroxide solution. The final concentrations of polymyxin B were 2 mg/mL, 1 mg/mL, and 0.5 mg/mL, while the final concentrations of hydrogen peroxide were 4 mmol/L, 2 mmol/L, and 1 mmol/L. After incubation at 37°C with shaking for 1 h, 900 μL of PBS was added to each mixture, followed by serial 10-fold dilutions. The diluted suspensions were plated on BA plates and incubated at 37°C for 3–5 days before bacterial colonies were counted. The PBS-treated group served as the negative control. The bacterial survival rate was calculated as follows: Survival rate (%) = (CFU treated by stress factors/CFU treated by PBS) × 100%.

To assess bacterial susceptibility to acidic conditions, 50 μL of bacterial suspension (5 × 10^5^ CFU/mL) was mixed with 950 μL of BB medium adjusted to different pH levels (4.5, 5.5, and 6.5). The mixtures were incubated at 37°C with shaking for 2 h, serially diluted 10-fold with PBS, and plated on BA plates. After incubation at 37°C for 3–5 days, bacterial colonies were enumerated. The group treated with neutral BB medium (pH = 7.2) served as the control, and the survival rate was calculated as follows: Survival rate (%) = (CFU treated by low pH BB/CFU treated by neutral BB) × 100%.

To determine bacterial sensitivity to SDS, 100 μL of bacterial suspension adjusted to OD_600_ = 1.0 was spread evenly onto BA plates. A sterile blank susceptibility disk was placed in the center of each plate, and 7 μL of 20% SDS solution was applied to the disk. Each test was performed in triplicate. The plates were incubated at 37°C for 3–4 days, and the diameter of the inhibition zones was measured.

To evaluate bacterial susceptibility to SNP, BA plates containing different concentrations of SNP were prepared, with final concentrations of 0.5 mM, 1 mM, and 2 mM SNP. Bacterial suspensions were adjusted to OD_600_ = 1.0 and serially diluted 10-fold with sterile PBS. Then, 2 μL of each dilution was spotted onto SNP-containing BA plates. The plates were incubated at 37°C for 5–6 days. BA plates without SNP served as the negative control. Bacterial growth was observed and documented by photography.

### 4.5 Biotinylation of *Brucella* surface proteins

*Brucella* surface proteins were biotinylated using EZ-Link Sulfo-NHS-Biotin (Thermo Fisher) according to the manufacturer's instructions. Briefly, bacterial cells (approximately 5 × 10^9^ CFU) were washed three times with ice-cold PBS (pH 8.0) to remove culture media and resuspended in 1 mL PBS. Then, 1 mg of Sulfo-NHS-Biotin reagent was added to the cell suspension. The mixture was incubated at room temperature for 30 min. Subsequently, the cells were washed three times with PBS containing 100 mM glycine to quench the reaction and remove excess biotin reagent and byproducts. The labeled cells were analyzed by SDS-PAGE and Western blot.

### 4.6 SDS-PAGE and Western blot analysis

*Brucella* samples, either non-labeled or biotin-labeled, were mixed with 5 × SDS loading buffer (0.1 M Tris buffer, pH 6.8, 4% SDS, 0.2% β-mercaptoethanol, 40% glycerol, and 0.002% bromophenol blue) and boiled for 10 min. The samples were then separated by SDS-PAGE on 12.5% resolving gels. Western blot analysis for LPS was performed as previously described (Yin et al., 2025). After electrophoresis, proteins or LPS were transferred to a nitrocellulose membrane. The membrane was blocked with 5% skim milk in PBS at room temperature and subsequently incubated with primary antibodies (mouse anti-O-antigen monoclonal antibody A76/12G12/F12, mouse anti-O-core monoclonal antibody A68/24D8/G9 or rabbit anti-GroEL polyclonal antibody) in PBST (PBS containing 0.5% Tween-20) at room temperature. After washing with PBST, the membrane was incubated with IRDye 680RD goat anti-mouse antibody or IRDye 800CW donkey anti-rabbit antibody (LI-COR, Lincoln, NE, USA, Cat. # 926-68070 and 926-32213) at a dilution of 1:20,000. For detection of biotin-labeled proteins, the membrane was directly incubated with IRDye 800CW streptavidin (LI-COR, Cat. # 926-32230) at a dilution of 1:2,500. All blots were washed with PBST between steps. Images were acquired by scanning using Odyssey Imaging System (LI-COR).

### 4.7 Fluorescent dye uptake

*Brucella* M5 and its derived strains were cultured in BB, PE or PE supplemented with purines medium to the mid-logarithmic phase. Cells were harvested by centrifugation at 8,000 rpm and 4 °C for 10 min, washed twice with sterile PBS, and resuspended to an OD_600_ of 1.0. Then, 200 μL of the bacterial suspension was transferred to each well of a 96-well black clear-bottom plate (Corning, NY, USA), with 3–5 replicates per strain. The following fluorescent dyes were applied accordingly: 2 μL of PI (1 mM) was added per well to assess membrane integrity, and fluorescence was measured immediately at Ex/Em = 535/617 nm every 2 min for 70 min; 2 μL of NPM (1 mM) was introduced per well to evaluate outer membrane permeability, with readings taken every 30 s for 600 s at Ex/Em = 355/460 nm; and 2.5 μL of NR (5 mM) was added per well to monitor lipid accumulation, and fluorescence was recorded at Ex/Em = 552/636 nm every 2 min over 70 min.

### 4.8 Cell infection assay

RAW264.7 murine monocyte-macrophage cells (TIB-71, ATCC) or human epithelial-like HeLa cells (CCL-2, ATCC) were seeded in 24-well cell culture plates at densities of approximately 2.5 × 10^5^ cells per well for RAW264.7 and 7 × 10^4^ cells per well for HeLa, respectively. Cells were cultured at 37 °C under 5% CO_2_ until a confluent monolayer formed, then washed twice with PBS, and incubated with 500 μL of DMEM per well.

Bacteria were grown in BB, PE, or PE supplemented with purines to the logarithmic phase and used to infect cells at a multiplicity of infection (MOI) of 100:1. To ensure synchronized infection, the inoculated plates were centrifuged at 400 × g for 5 min at room temperature. After 1 h of infection at 37 °C, cells were washed three times with PBS and lysed with 200 μL of 0.25% Triton X-100 in PBS. The lysates were serially diluted 10-fold and plated on BA plates to determine the number of adherent bacteria (CFU).

To quantify internalized bacteria, after the initial washing step, cells were treated with DMEM containing 100 μg/mL gentamicin for 1 h at 37 °C to eliminate extracellular bacteria. The cells were then washed three times with PBS, lysed with 0.25% Triton X-100, and the lysates were diluted and plated as described above to enumerate intracellular bacteria.

To assess intracellular survival, following the elimination of extracellular bacteria, the cells were maintained in DMEM supplemented with 50 μg/mL gentamicin and 2% FBS. At 1, 8, 24, and 48 h.p.i., the medium was removed, and the cells were washed three times with PBS before lysis with 0.25% Triton X-100. The lysates were serially diluted and plated on BA plates to determine bacterial CFU counts.

To evaluate the effect of purine synthesis on intracellular survival of the *purD* deletion strain, exogenous purines-−1 mM guanine, 1 mM adenine, and 1 mM hypoxanthine (Sangon Biotech)—were added to the maintenance DMEM where indicated.

### 4.9 Indirect immunofluorescence assay

RAW264.7 cells were cultured on 14 mm glass coverslips (NEST, Wuxi, China) pre-placed in 24-well plates and infected following the aforementioned protocol. Where indicated, the cell maintenance medium was supplemented with 1 mM guanine, 1 mM adenine, or 1 mM hypoxanthine. At 24 h.p.i., cells were washed three times with PBS containing 0.05% Tween-20 (PBST) and fixed with 4% formaldehyde for 15 min at room temperature. After three PBST washes, cells were permeabilized with 0.5% Triton X-100 in PBS for 15 min at room temperature and blocked with 5% BSA in PBS for 30 min at 37 °C.

The coverslips were then incubated sequentially with rabbit anti-*Brucella* primary antibody (1:1000 dilution) (Yin et al., 2023) and Alexa Fluor 488-conjugated goat anti-rabbit secondary antibody (1:500 dilution; Thermo Fisher Scientific, Waltham, MA, USA, Cat. # A-11008) for 1 h each at 37 °C, with an intermediate blocking step using 5% BSA-PBS for 30 min at 37 °C. Subsequently, samples were stained with CoraLite^®^594-conjugated anti-mouse LAMP1 antibody (1:100 dilution; Proteintech, Wuhan, China, Cat. # CL594-65050,) for 1 h at 37 °C.

Following three PBST washes, nuclei were stained with 2 μg/mL DAPI (Beyotime, Suzhou, China) for 2 min. After three additional PBST washes and a final rinse with deionized water, coverslips were air-dried and mounted using ProLong™ Glass Antifade Mountant (Thermo Fisher Scientific). Images were acquired using a Zeiss Axio Imager 2 upright fluorescence microscope (Carl Zeiss Microscopy GmbH, Jena, Germany) with a 100 × oil immersion objective.

Acquired images were processed using Adobe Photoshop 2023 (version 24.3; Adobe Inc., San Jose, CA, USA), and the percentage of LAMP1-positive *Brucella* was quantified by examining at least 100 bacteria per sample.

### 4.10 Mouse infection experiments

To evaluate the virulence of *Brucella*, this study employed both high-dose and low-dose infection models in mice to assess survival rates and splenic bacterial burden, respectively. The detailed procedures were as follows:

*Brucella* M5 and its derivative strains were cultured in BB medium to the logarithmic growth phase. The bacterial suspension was adjusted to an OD_600_ of 1.0 (approximately 5 × 10^9^ CFU/mL) and diluted with PBS to the required concentrations for infection.

For the survival assay, eighteen 6- to 8-week-old specific pathogen-free (SPF) Balb/c mice were randomly divided into three groups (*n* = 6 per group): the parental strain group, the mutant strain group, and the complemented strain group. Each mouse was intraperitoneally inoculated with 0.1 mL of bacterial suspension containing 2.5 × 10^8^ CFU. Survival was monitored daily for 14 days.

To evaluate splenomegaly and bacterial load, forty SPF Balb/c mice (6–8 weeks old) were equally assigned to four groups (*n* = 10 per group): parental strain infection, mutant strain infection, complemented strain infection, and PBS control. Each mouse received an intraperitoneal injection of 0.1 mL bacterial suspension containing 1 × 10^6^ CFU or PBS. At 2 and 4 w.p.i., five mice from each group were euthanized by cervical dislocation. Spleens were aseptically collected and weighed. Each spleen was homogenized in 3 mL of 0.25% Triton X-100 in PBS, serially diluted 10-fold, and plated on BA plates. The plates were incubated at 37 °C under 5% CO_2_ for 3–5 days before bacterial colonies were counted to determine splenic bacterial load.

### 4.11 Histopathological analysis

Liver tissues were collected from infected mice at 2 and 4 w.p.i. as described above. The tissues were fixed in 4% paraformaldehyde for 24 h and subsequently sent to Wuhan Borf Biological Technology Co., Ltd. (Wuhan, China) for paraffin embedding and hematoxylin-eosin (H&E) staining. Whole-slide images (WSIs) were acquired with a Pannoramic MIDI II system (3DHistech, Budapest, Hungary), and image analysis was performed with CaseViewer v2.4 (3DHistech). For each group, five randomly selected regions of 15 mm^2^ per section were analyzed to quantify the number of granulomas per unit area. Additionally, 60 granulomas were randomly chosen from each group for diameter measurement, followed by statistical analysis.

### 4.12 Statistical analysis

Statistical analyses were performed using GraphPad Prism version 9.5 (GraphPad Software, San Diego, CA, USA). Comparisons between two groups were conducted using Student's *t*-test. For comparisons among multiple groups, one-way or two-way analysis of variance (ANOVA) was applied, followed by Dunnett's multiple comparison test. A *p*-value of less than 0.05 was considered statistically significant.

## 5 Conclusions

This study aimed to investigate the role of *de novo* purine synthesis in the pathogenicity of *B. melitensis*. The results indicate that impairment of purine synthesis compromises membrane homeostasis in *Brucella*, enhances bacterial lipid biosynthesis, and reduces its ability to invade host cells, survive intracellularly, cause mortality in mice, colonize the spleen, induce splenomegaly, and form granulomas in the liver. These findings provide important clues for the development of attenuated purine-auxotrophic vaccines against *Brucella*.

## Data Availability

The original contributions presented in the study are included in the article/Supplementary material, further inquiries can be directed to the corresponding authors.
