# Supplementary material for: Disruption of purine de novo synthesis pathway impairs membrane homeostasis, intracellular survival, and virulence of Brucella melitensis
Source: Front Microbiol. 2025 Dec 16;16:1721961. doi: 10.3389/fmicb.2025.1721961 (PMC12748177; doi:10.3389/fmicb.2025.1721961)
Supplement: Supplementary file 1 [file Data_Sheet_1.pdf]

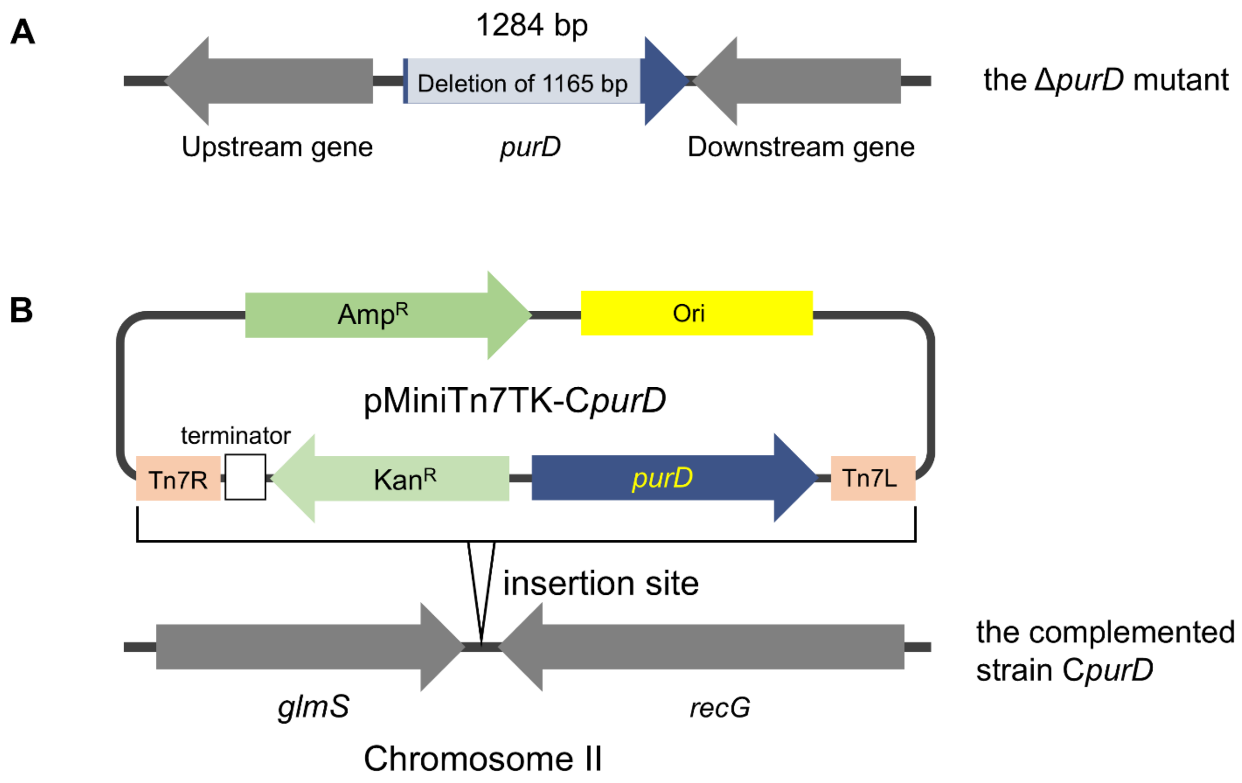

Figure S1. Schematic diagram of the Δ*purD* mutant and complemented strain *CpurD*. (A) Construction strategy of the Δ*purD* mutant. (B) Construction strategy of the complemented strain *CpurD*.

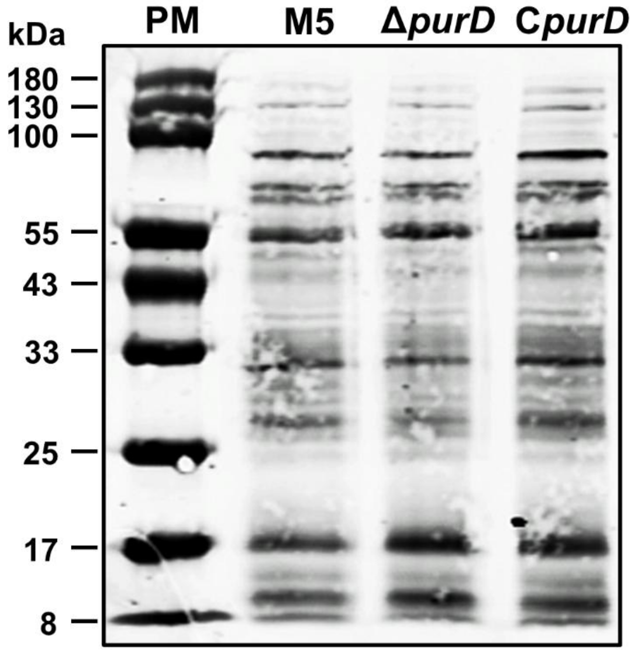

Figure S2. Western blot analysis of biotin-labeled outer membrane proteins in *Brucella*.

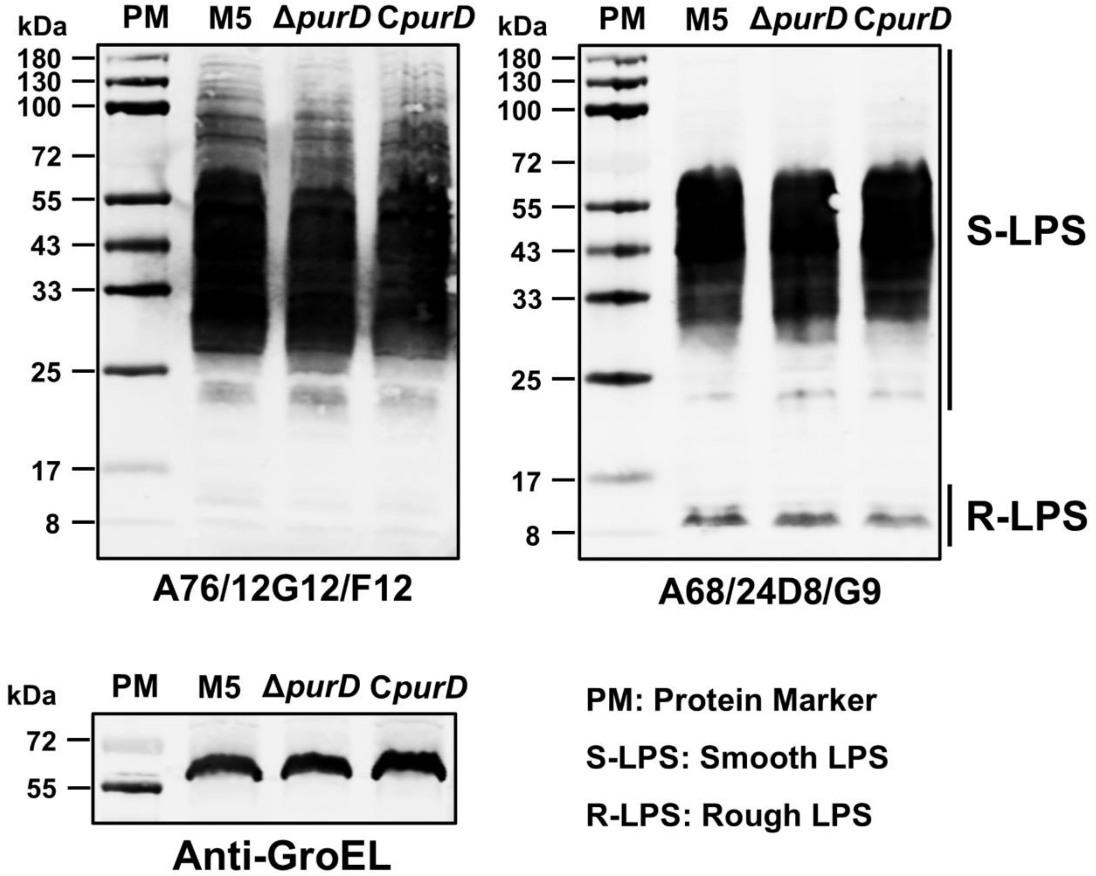

Figure S3. Western blot analysis of LPS expression in *Brucella*.
